# Preschool-aged children recognize ambivalence: emerging identification of concurrent conflicting desires

**DOI:** 10.3389/fpsyg.2015.00425

**Published:** 2015-04-10

**Authors:** Kristin Rostad, Penny M. Pexman

**Affiliations:** Language Processing Laboratory, Department of Psychology, University of Calgary, Calgary, AB, Canada

**Keywords:** ambivalence, conflicting desires, approach/avoidance, ulysses conflict, desire reasoning

## Abstract

We examined the ability of preschool-aged children to identify conflicting, or ambivalent, desire states (e.g., “I want to go to the birthday party because there will be cake, but I also don’t want to go because I’m having fun playing at home”). Participants were 4- and 5-year-old children, and a group of undergraduate students (*n* = 20 in each age group). They were presented with 14 scenarios involving both “single desire” and “dual desire” states, including both approach (i.e., “want”) and avoidance (i.e., “not want”) desires. Our primary interest was children’s ability to identify concurrent conflicting “dual desire” states, and this ability was found in most of the 5-year-old age group tested and in about half of the 4-year-old age group. As such, these results provide evidence that children can identify ambivalence at earlier ages than previously reported. In addition, results showed that the challenge in recognizing ambivalence is the presence of desires of opposite valence directed at the same target.

## Introduction

Ambivalence is the state of both wanting and not wanting something, such as when you are tempted by a late-afternoon snack but at the same time don’t want to spoil your appetite for dinner. This is a common mental state, and one that is sometimes referred to as a *Ulysses conflict*, in reference to Ulysses’ ambivalence over wanting to hear the songs of the Sirens but not wanting to meet the fate of those who did (Elster, 1979).

The experience of concurrent conflicting desires is something adults can typically identify and reason about, grasping the duality and conflicting nature of these situations. We know less, however, about when children can identify these ambivalent desire scenarios. Previous research has demonstrated that between 3 and 4 years of age, children begin to learn that a single scenario can produce conflicting desires in different individuals (Flavell et al., 1992; Rakoczy et al., 2007). Specifically, they can appreciate that while Person A might have desire “X,” Person B might have desire “Y.” Research by Bennett and Galpert (1993) demonstrated that as early as age 5 children understand that there can be an experience of conflicting desires within one individual, however, this understanding is limited to successive desires (i.e., desire “X” followed by desire “Y”). In a follow up experiment involving concurrent conflicting desires, such as a character being invited to two birthday parties on 1 day, only 11-year old children asserted that the character could, at the same time, both want and not want to go to the party. As such, the recognition that it is possible to have concurrent conflicting desires within an individual seems to be particularly challenging.

More recent research on concurrent conflicting desire states examined identification in 4- to 7-year-old children and a group of adults (Choe et al., 2005). In Study 1, participants were shown a video depicting one of three scenarios: two individuals with conflicting desires, one individual with successive conflicting desires, or one individual with concurrent conflicting desires (“Ulysses condition”). In the concurrent conflicting desires condition, the script conveyed the want desire, and the actor’s body language and facial expressions (longing glances, hesitation) conveyed their conflict. Study 1 involved a verbal open-ended response format, and only the adult participants provided responses which referred to a conflicting mental state. In Study 2, the same age groups were tested but only the Ulysses condition was included. The scenarios were depicted using still images from the Study 1 video materials and narration described the character’s facial expressions and hesitation (e.g., Jane saw the cookies on the table. She smiled at first, but in a little while, she frowned…). The response format was modified such that participants simply had to identify the characters’ mental state (e.g., What was Jane’s mind like?) from three options depicted in thought bubbles: I want to eat these cookies, I don’t want to eat these cookies, or I both want and not want to each these cookies at the same time. Using this response format, both the adults and the 7-year-old children were able to identify concurrent conflicting desire states (i.e., they selected the third thought bubble option at an above-chance level). The multiple-choice thought-bubble response format in Study 2 appeared to be easier for the child participants to understand than the open-ended response format in Study 1, which is consistent with previous research (Harter and Whitesell, 1989).

Although the adult participants in Choe et al.’s (2005) Study 2 performed above chance (65% accuracy), they were actually less accurate than in Study 1 (85% accuracy). The procedure in both studies provided only minimal cues about the mental state of the character, such as whether she was smiling or frowning, and the verbal script provided few details. In addition, the scenarios included themes that were both familiar (wanting cookies) and unfamiliar (wanting to smoke cigarettes) to children. Choe et al. (2005) concluded that children’s ability to recognize concurrent conflicting mental states is emerging at age 7. They noted that this was somewhat surprising, given that theory of mind skills develop earlier, but suggested the delay could be attributed to the fact that there are usually few outward cues that a person is experiencing conflicting mental states. In the absence of such cues, children struggle “due to the lack of experience with internal conflicts of their own.” (p. 392).

In a subsequent study, Rostad and Pexman (2014) directly assessed the relationship between children’s theory of mind skills and their ability to identify conflicting desires. In addition, Rostad and Pexman (2014) investigated whether, if children were given more insight about the speaker’s desires, concurrent conflicting desire identification could be found in even younger age groups. Child participants ranged in age from 4 to 7 years, and a group of adults were included for comparison. There were four testing conditions: “Approach” involved a single “want” desire, “Avoidance” involved a single “not want” desire, “No Conflict” involved dual desires which were not mutually exclusive (i.e., wanting “X” and not wanting “Y”), and “Conflict” involved dual desires which were mutually exclusive and created ambivalence (i.e., wanting “X” and not wanting “X”). The testing procedure involved child-friendly characters and desire items, and information about the character was provided to give context to the desire state(s): This is David. David wants to ride a bicycle right now because there is a big hill near his house that he likes to ride down really fast. David also does not want to ride a bicycle right now because the last time he rode a bicycle he fell and hurt his knee. As in Study 2 of Choe et al. (2005), a multiple-choice thought-bubble response format was employed.

The results of the Rostad and Pexman (2014) study showed, first, that children’s identification of conflicting desires was related to their theory of mind (second order false belief) and executive function (Dimensional Change Card Sort, DCCS, Zelazo, 2006) skills. Second, and consistent with the results of Choe et al. (2005), Rostad and Pexman (2014) found that adults and 7-year-old children were able to correctly identify concurrent conflicting desire states in the “Conflict” condition (100 and 88% accuracy, respectively). However, the 6-year-olds in Rostad and Pexman (2014) also performed at significantly above-chance levels in the “Conflict” condition (83% accuracy), and the 5-year-olds were approaching above-chance levels (52% accuracy, in a task where chance was 33%). The 4-year-olds did not show accurate identification for desire states in the “Conflict” condition, and were significantly below chance in selecting the conflicting desire response option (13% accuracy).

### Current Experiment

It appears, therefore, that children as young as 6 years of age, and even some 5-year-olds, may be able to identify ambivalence and that the ability to identify ambivalence parallels development of theory of mind and executive function skills (Rostad and Pexman, 2014). The current experiment was conducted to further examine this issue, and had two main goals. The first goal was to examine whether further modifications to the testing procedure would allow children to find success in the “Conflict” condition at even younger ages. In particular, we focused on modifications that have been shown to reduce the executive functioning and other demands in mental state reasoning tasks. Rubio-Fernández and Geurts (2013) showed that 3-year-olds could pass a false belief task when the procedure was modified to help children take and track the perspective of the protagonist. In the current experiment, we modified the procedure to help children take the speaker’s perspective: we presented a puppet as speaker in each story, and used a response procedure that involved the children placing the appropriate desires (response objects) in the puppet’s mind (thought bubble) themselves. False belief performance is also supported by language (labeling) that highlights the protagonist’s perspective (e.g., Low and Simpson, 2012). In the current experiment, we provided children with additional description of the basis for characters’ desires on the assumption that this could help them represent the characters’ conflict. Finally, working memory demands are one of the challenges children face in the false belief task (e.g., Carlson et al., 2002) and we sought to reduce those in the current experiment by eliminating the use of a long and verbally complex response prompt. That is, recall that in both the Choe et al. (2005) and Rostad and Pexman (2014) studies children chose a thought bubble only after each bubble had been re-described and a response invited. Here the prompt was reduced from an average of 40 words per trial to 14, and the particularly complex phrases were eliminated.

A second goal of the present study was to examine more precisely the nature of children’s “dual desire” identification, to determine what is difficult about ambivalence specifically. Previous research showed that children find avoidance (“not want”) desires more difficult to identify than approach (“want”) desires (e.g., Fritzley and Lee, 2003; Rostad and Pexman, 2014). Here we investigated whether this negation is part of what makes ambivalence challenging for children. That is, is the challenge of ambivalence related to the presence of avoidance desires regardless of the mutual exclusivity of those desires (that is, even if the desires are not directed to the same target?), or is mutual exclusivity an important part of the challenge of ambivalence? The current experiment was designed to adjudicate between these possibilities by examining children’s identification of a variety of “dual desire” scenarios.

Given that 6- and 7-year-old children in Rostad and Pexman (2014) performed above chance, only 4- and 5-year-old children were included in the current experiment. A group of adult participants was also included for comparison.

## Materials and Methods

### Participants

Twenty participants were included in each of the three age groups: 4-year-olds (*M* = 4.50 years, SD = 0.15, range = 4.16–4.77), 5-year-olds (*M* = 5.52 years, SD = 0.24, range = 5.13–5.94), and adults (*M* = 21.83 years, SD = 1.77, range = 18.29–24.57). Child participants were recruited through the University of Calgary ChILD participant database. They were typically-developing, primarily of Caucasian descent, and resided in families from mostly middle- to upper-middle socioeconomic classes. There were equal numbers of male and female participants in both of the child age groups. The adult participants were undergraduate students. There were 17 females and 3 males in the adult group, which is representative of the local undergraduate Psychology population. Adult participants received one credit for their participation (which equates to 1%), and this credit could be applied toward a Psychology course of their choice. Eight additional children participated but were excluded for reasons described below, including a response pattern during the desire appreciation task (*n* = 3), failure to pass the “single desire” criterion (*n* = 2), failure to meet the training criterion (*n* = 2), and not completing the testing protocol (*n* = 1).

The University of Calgary Conjoint Faculties Research Ethics Board approved this research project. Child participants gave verbal assent and their parent or guardian gave written informed consent for the child to participate. Adult participants gave written informed consent.

### Materials

**FIGURE 1:**
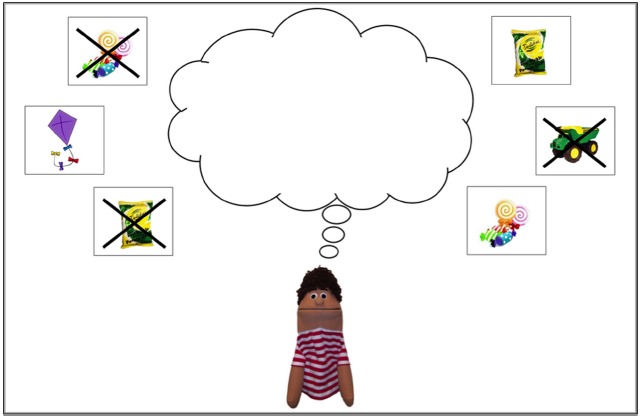
**Example of test display for the candy story theme, with main character, thought bubble, and response image magnets**.

The materials for the desire appreciation task included 14 different test displays (see Figure 1 for an example). Each test display was mounted on a metal sheet and contained the image of a puppet with an empty thought bubble. Surrounding the empty thought bubble were six different response options on magnets. Three of the response options were “want” desires and three were “not want” desires (indicated by an image crossed out with an “X”). Four of the six response images were related to the theme(s) introduced during the script for that trial and the other two images were distracters. An easel was used to present the test display. The easel was positioned on the testing table in front of the participant but out of arm’s reach. Testing materials also included 14 different puppets, one for each of the experimental trials.

### Procedure

Participants were tested individually, while seated at a table across from the experimenter. The desire appreciation task began with a training procedure to familiarize participants with thought bubbles and the response options. Participants were given brief instructions about what thought bubbles represent [i.e., “thought bubbles show what (the character) is thinking”] and about what the response options represent [i.e., “putting this picture in the thought bubble would mean that (the character) wants ice cream”]. They had the opportunity to practice placing response options in the thought bubble for a series of training stories. Some of the training stories involved single desires and some involved dual desires, but none involved concurrent conflicting desires. Participants were given corrective feedback about their response selections during the training trials. Participants were not given any corrective feedback about their response selections during the testing trials.

At the beginning of each of the 14 experimental trials, the experimenter introduced a hand puppet. The experimenter then read a script containing information about the desire(s) of the puppet. After reading the script, the experimenter moved the test display to the table and said “Remember everything I told you about (puppet). Now show me what (puppet) is thinking.” At this point the participant selected response option magnets and moved them into the thought bubble. Two trials were provided in each condition, with the exception of the “Conflict” condition which had four trials. Given that the “Conflict” condition was the primary condition of interest, it was decided that two additional trials would allow for more confidence in the results. Increasing each of the other conditions to four trials would have significantly lengthened the procedure.

### Design

The study involved a within-subjects design and six different experimental conditions. As such, the information in each script was based on one of the following six test conditions. In the “Approach” condition, the puppet had a single “want” desire (e.g., wants to eat candy). In the “Avoidance” condition, the puppet had a single “not want” desire (e.g., does not want to eat candy). In the “Approach/Approach” condition, the puppet had two “want” desires regarding two different stimuli (e.g., wants to eat candy and also wants to drink pop). In the “Avoidance/Avoidance” condition, the puppet had two “not want” desires regarding two different stimuli (e.g., does not want to eat candy and also does not want to eat chips). In the “No Conflict” condition, the puppet had a “want” desire regarding one stimulus and a “not want” desire regarding a second stimulus (e.g., wants to eat candy and also does not want to eat chips). Finally, in the “Conflict” condition, the puppet had mutually exclusive “want” and “not want” desires regarding the same stimulus (e.g., wants to eat candy and also does not want to eat candy). Table 1 contains a full example of the testing scripts for one theme. Each trial included a different puppet character and a different story theme.

**TABLE 1:** **Example of desire appreciation conditions using the candy story theme**.

“Approach” condition	“Avoidance” condition	“Approach/Approach” condition
This is David. David wants to eat candy right now because his sister brought home a bag of candy from school and it has David's favorite candy in it. David's favorite candy are lollipops, and he really likes the strawberry ones.	This is Karen. Karen does not want to eat candy right now because she got a tummy ache the last time she ate candy. Karen's tummy ache lasted for 2 days, and she still remembers how bad it felt.	This is Alex. Alex wants to eat candy right now because his sister brought home a bag of candy from school and it has Alex's favorite candy in it. Alex's favorite candy are lollipops, and he really likes the strawberry ones. Alex also wants to drink pop right now because his dad just brought home orange pop from the grocery store. Orange pop is Alex's favorite pop, so it's a special treat.
**“Avoidance/Avoidance” condition**	**“No Conflict” condition**	**“Conflict” condition**
This is Brenda. Brenda does not want to eat candy right now because she got a tummy ache the last time she ate candy. Brenda's tummy ache lasted for 2 days, and she still remembers how bad it felt. Brenda also does not want to eat chips right now because they only have dill pickle and that is the flavor she hates the most. Brenda doesn't like real pickles either, but hates dill pickle flavored chips even more.	This is Martin. Martin wants to eat candy right now because his sister brought home a bag of candy from school and it has Martin's favorite candy in it. Martin's favorite candy are lollipops, and he really likes the strawberry ones. Martin also does not want to eat chips right now because they only have dill pickle and that is the flavor he hates the most. Martin doesn't like real pickles either, but hates dill pickle flavored chips even more.	This is Michelle. Michelle wants to eat candy right now because her sister brought home a bag of candy from school and it has Michelle's favorite candy in it. Michelle's favorite candy are lollipops, and she really likes the strawberry ones. Michelle also does not want to eat candy right now because she got a tummy ache the last time she ate candy. Michelle's tummy ache lasted for 2 days, and she still remembers how bad it felt.

### Scoring

Responses on each trial of the desire appreciation task were scored dichotomously (“0” for incorrect and “1” for correct). For an item to be scored as correct in the “dual desire” conditions, the participant had to select both of the correct response images and no other images. All other answers were scored as incorrect. Proportion correct scores were calculated for each participant in each of the six conditions.

There were three exclusion criteria regarding performance on the desire appreciation task. First, participants were excluded if they did not meet the training criterion of selecting at least 4/8 response images correctly during the training trials. Given that participants were provided with corrective feedback, it was considered quite problematic if participants did not successfully learn the task. Second, participants were excluded if they did not meet the “single desire” criterion. This was defined as a score of at least 1/4 on the “Approach” and “Avoidance” conditions during testing. The “Approach” and “Avoidance” conditions were designed to be very straightforward and easy to understand; not answering those questions correctly was taken as a sign that the task was not understood.

Finally, participants were excluded if they displayed a particular response pattern during the desire appreciation trials. Specifically, three of the child participants demonstrated a response strategy of placing both the “want” and “not want” response images in the thought bubble for all themes mentioned during the script. In the “Approach/Approach,” “Avoidance/Avoidance,” and “No Conflict” conditions, this meant that four images were placed in the thought bubble, which was scored as incorrect. The “Approach” and “Avoidance” conditions were also scored as incorrect using this strategy, because both the “want” and “not want” images for the story theme were placed in the thought bubble. However, in the “Conflict” condition, this strategy led to a correct response (i.e., the “want” and the “not want” image for the one story theme were placed in the thought bubble). This exclusion criterion was established because participants who used this strategy had very high proportion correct scores in arguably the most challenging “Conflict” condition, and scores of zero in all other conditions. There were no adult participants who needed to be excluded for any of the three exclusion criteria.

## Results

**FIGURE 2:**
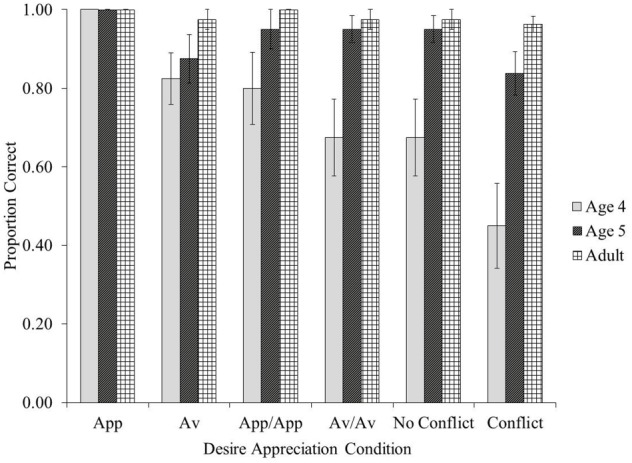
**Proportion correct scores for the desire appreciation task as a function of condition and age group**. App = “Approach” condition. Av = “Avoidance” condition. App/App = “Approach/Approach” condition. Av/Av = “Avoidance/Avoidance” condition. Bars represent standard errors.

There were no significant effects of gender or testing order on accuracy in any of the age groups (all *ps* > 0.05), so these variables were collapsed for all further analyses. Figure 2 displays the results for all participants in each of the six desire appreciation conditions. As can be seen in Figure 2, the adults demonstrated very high accuracy in each of the six conditions (96% accuracy or better). This suggests that adults could readily interpret the stories as intended. Given that the performance of the child participants was of greatest interest, only the children’s data were included for the remainder of the analyses.

### Chance Analyses

Performance by both of the child age groups in each of the six testing conditions was compared to chance using one-sample *t*-tests (two-tailed). Chance (0.10) was calculated based on one or two items being selected at random from the four related theme images (i.e., there were 10 possible responses with one or two non-distracter items, one of which was the correct response). Both child age groups performed at significantly above-chance levels in all six testing conditions (all *ps* < 0.004).

Although the 4-year-olds were performing at an above-chance level even in the most challenging “Conflict” condition, examination of the frequency data in that condition revealed that performance was quite bimodal, with half of the 4-year-olds (*n* = 10) unable to accurately identify desires (i.e., proportion correct scores of 0.00) and almost half (*n* = 8) demonstrating accurate identification (i.e., proportion correct scores of 1.00). The remaining participants (*n* = 2) were precisely in the middle (i.e., proportion correct scores of 0.50). For the 5-year-old age group, the data was more skewed toward accurate performance. That is, one participant had a proportion correct score of 0.00, one had a proportion correct score of 0.50, and the remaining 18 had proportion correct scores of either 0.75 or 1.00.

### Condition Effects

The main effect of condition (collapsed across the child age groups) was significant [*F_r_*_(5)_ = 38.51, *p* < 0.001], suggesting that there were differences in children’s accuracy between the desire appreciation conditions. Bonferroni-Holm-corrected α levels were used for follow-up comparisons, and the *p* values were converted for reporting purposes using the Aickin and Gensler (1996) method. Given that there were six conditions in the desire appreciation task, 15 follow-up tests would have been required to examine every pair of conditions. Therefore, planned comparisons were performed on the seven pairs of comparisons of greatest interest.

First, children’s accuracy in the “Approach” condition was compared to that in each of the other five thought bubble conditions. Accuracy in the “Approach” condition (*M* = 1.00) was significantly higher than in the “Avoidance” condition (*M* = 0.85, *T* = 2.97, *p* = 0.008, *r* = 0.47), “Avoidance/Avoidance” condition (*M* = 0.81, *T* = 2.88, *p* = 0.012, *r* = 0.46), “No Conflict” condition (*M* = 0.81, *T* = 2.88, *p* = 0.012, *r* = 0.46), and “Conflict” condition (*M* = 0.64, *T* = 4.10, *p* < 0.001, *r* = 0.65). These results are consistent with the findings from Rostad and Pexman (2014), and indicate that single “want” desires are relatively easy for children to identify. There was no significant difference between the “Approach” and “Approach/Approach” conditions (*M* = 0.88, *T* = 2.24, *p* = 0.126, *r* = 0.35), suggesting it is not necessarily more difficult for children to identify dual desires than single desires.

Next, children’s accuracy in the “Avoidance” and “Avoidance/Avoidance” conditions was compared, and no significant difference was found (*T* = 0.61, *p* = 0.627, *r* = 0.10). This further indicates that “dual desire” conditions are not inherently more difficult than their “single desire” counterparts. Finally, children’s accuracy in the “No Conflict” and “Conflict” conditions was compared, and the “No Conflict” condition was found to be significantly easier than the “Conflict” condition (*T* = 2.71, *p* = 0.018, *r* = 0.43). This suggests that it is the mutual exclusivity of the “want” and “not want” desires in the “Conflict” condition which presents a challenge for children, and not simply the presence of opposite-valence desires.

### Age Effects

Mann-Whitney *U*-tests were conducted to compare performance by the 4- and 5-year-olds in each of the six thought bubble conditions. In the “Approach,” “Avoidance,” and “Approach/Approach” conditions, there were no significant effects of age group (*U* = 200.00, *p* = 1.00; *U* = 181.00, *p* = 0.657, *r* = 0.11; *U* = 170.00, *p* = 0.342, *r* = 0.22; respectively). However, there was a significant age effect in each of the “Avoidance/Avoidance,” “No Conflict” and “Conflict” conditions, with the 5-year-olds consistently outperforming the 4-year-olds (*U* = 135.00, *p* = 0.021, *r* = 0.37; *U* = 135.00, *p* = 0.021, *r* = 0.37; *U* = 124.00, *p* = 0.029, *r* = 0.35; respectively).

## Discussion

The purpose of the current experiment was to examine the identification of concurrent conflicting desires in preschool-aged children. Concurrent conflicting desires were defined as the presence of two desire states that were mutually exclusive (e.g., wanting to ride a bicycle and not wanting to ride a bicycle at the same time). In previous research Choe et al. (2005) found that it was not until age 7 that children could accurately identify concurrent conflicting desires. In a subsequent study, Rostad and Pexman (2014) provided children with additional explanation about the speaker’s desires and found that children as young as six could recognize conflicting desires. However, additional limitations with previous testing procedures may have prevented younger children from demonstrating a higher level of accuracy.

The procedure used in the current experiment involved a number of modifications to previous testing procedures designed to reduce the demands of the task. Multiple contextual sentences were included to provide more motivation and explanation for each desire state. We introduced puppets and active selection of response options for the speaker’s “mind” to help children track the speaker’s perspective. To reduce the working memory demands of the testing procedure we also shortened the test question. With these modifications, the 4- and 5-year-old children who participated in the current experiment were able to demonstrate significantly above-chance performance in each of the six desire appreciation conditions including the “Conflict” condition, which represented concurrent conflicting desires. Specifically, the 5-year-olds performed with 84% accuracy in the “Conflict” condition, and the 4-year-olds performed with 45% accuracy. Our goal was to identify the age at which children begin to identify concurrent conflicting desires; we did not compare these modifications systematically across multiple experiments, so we are not able to identify which particular modification(s) were responsible for the earlier accuracy in the current study.

In addition, the relative difficulty of “dual desires” was elucidated in the current experiment by the inclusion of four different “dual desire” conditions. Results showed that the “Conflict” condition was the most difficult for child participants and was significantly more challenging than the “No Conflict” condition, which also included one “want” desire and one “not want” desire. These results suggest that the challenge of the “Conflict” condition was not simply that it involved the representation of two concurrent desire states. If that were the case, all four of the “dual desire” conditions would have been equally difficult to identify. It also cannot be argued that the challenge of the “Conflict” condition is that it requires representation of two concurrent opposite-direction desire states. If that were the case, performance in the “Conflict” condition would have been comparable to performance in the “No Conflict” condition. Rather, it appears fair to conclude that the “Conflict” condition is the most challenging because it involves the presence of one “want” desire and one “not want” desire which are mutually exclusive (representing an ambivalent desire state). Thus, the challenge of ambivalence seems to be representation of opposite desires toward the same object.

In the present study, we observed that there were individual differences in the 4-year-old group, with about half demonstrating accurate identification in the “Conflict” condition. These individual differences could involve the related constructs of theory of mind and executive function skills (Rakoczy, 2010; Rostad and Pexman, 2014). Indeed, in order to provide an accurate response in the “Conflict” condition, we presume that children had to detect the conflict in the puppet’s opposite desires about the same object, and also had to recognize that conflict as a possible mental state. This would likely involve cognitive flexibility, and it is striking that 4-year-olds showed emerging identification of conflicting desires in the present study and the same age group shows intermediate performance on the DCCS task (Zelazo, 2006). Of course, it is also possible that, as Choe et al. (2005) speculated, children’s performance is related to their exposure to different beliefs about the relationship between mental states and behavior, and notions about the extent to which desires arise from internal or external forces. These are important issues for future research.

The results from the present experiment indicate that some children are able to identify concurrent conflicting desire states by 4 years of age. However, the conflicting desires we presented (i.e., eating candy, riding bicycles) were directed toward familiar concrete objects and events which probably do not evoke the same sort of abstract reasoning and complex emotional response as, for example, conflicting desires regarding living with one parent versus the other. The current results suggest that basic identification of rather simplistic conflicting desires states is possible even for 4-year-olds. For more complicated scenarios, particularly those involving mixed emotional states, other research indicates that appreciation for concurrent and opposite-valence emotions is not developing until approximately age 10 (Harter and Whitesell, 1989; see Bennett and Galpert, 1993, for further discussion about differences between emotions and desires).

The present results contribute to previous findings suggesting that there appears to be a developmental trend in children’s identification of desire states. That is, identification begins in early childhood with simple and single desire states (Flavell et al., 1992), continues on to dual but non-conflicting and/or non-concurrent desire states (Bennett and Galpert, 1993), and finally involves the dual and concurrent conflicting desire states examined in the current experiment. The present results show that even preschool-aged children can demonstrate this impressive cognitive achievement.

### Conflict of Interest Statement

The authors declare that the research was conducted in the absence of any commercial or financial relationships that could be construed as a potential conflict of interest.
